# Dietitians Australia position statement on telehealth

**DOI:** 10.1111/1747-0080.12619

**Published:** 2020-06-28

**Authors:** Jaimon T. Kelly, Margaret Allman‐Farinelli, Juliana Chen, Stephanie R. Partridge, Clare Collins, Megan Rollo, Rebecca Haslam, Tara Diversi, Katrina L. Campbell

**Affiliations:** ^1^ Menzies Health Institute Queensland, Faculty of Medicine Griffith University Gold Coast Australia; ^2^ Charles Perkins Centre, Discipline of Nutrition and Dietetics, School of Life and Environmental Sciences The University of Sydney Sydney Australia; ^3^ Westmead Applied Research Centre, Faculty of Medicine and Health The University of Sydney Sydney Australia; ^4^ Priority Research Centre in Physical Activity & Nutrition and School of Health Sciences, Faculty of Health and Medicine The University of Newcastle Callaghan Australia

**Keywords:** chronic disease, diet, digital health, nutrition, telehealth

## Abstract

It is the position of Dietitians Australia that clients can receive high‐quality and effective dietetic services such as Medical Nutrition Therapy (MNT) delivered via telehealth. Outcomes of telehealth‐delivered dietetic consultations are comparable to those delivered in‐person, without requiring higher levels of additional training nor compromising quality of service provision. Dietitians Australia recommends that policy makers and healthcare funders broaden the recognition for telehealth‐delivered dietetic consultations as a responsive and cost‐effective alternative or complement to traditional in‐person delivery of dietetic services. The successful implementation of telehealth can help to address health and service inequalities, improve access to effective nutrition services, and support people with chronic disease to optimise their diet‐related health and well‐being, regardless of their location, income or literacy level, thereby addressing current inequities.

## 1 BACKGROUND

Nutrition‐related chronic diseases are the leading cause of ill health in Australia.^1^ Within the next 5 years, it is estimated that over 75% of Australian adults will be living with overweight or obesity.^2^ One in two Australian adults have a chronic disease, with over seven million (35% of the Australian population) living with nutrition‐related chronic disease, including type 2 diabetes, cardiovascular disease, obesity, diet‐related cancer, chronic kidney disease and mental health conditions.^1^, ^3^ Australia, like many developed countries, has an ageing population, which presents a significant challenge for the healthcare system. Together these will drive up healthcare expenditure and present a multitude of additional societal, geographical and workforce challenges for the healthcare system to manage.^4^

Telehealth‐delivered nutrition consultations offer a flexible modality to provide effective and cost‐effective medical nutrition therapy (MNT), regular monitoring and support to the large numbers of people in the community requiring dietetic services, in particular those with obesity^5^ and nutrition‐related chronic disease.^6^, ^7^ According to the World Health Organisation, the term “telehealth” refers to the remote delivery of health services using information and communication technologies to exchange health information, either synchronously (ie, two‐way communication in real time; e.g. telephone and videoconference consultations) and/or asynchronously (ie, one way communication at any time; e.g. text‐messaging and web‐portals).^8^ Digital health modalities (encompassing telehealth) also include the remote delivery of nutrition interventions via electronic health (eHealth) modes, such as web‐based programs, software programs and a range of mobile health (mHealth) options, such as smartphone applications (apps), text messaging programs and wearable devices.

Many Australians cannot access dietetic services due to economic, geographic and sociographic barriers. There is a high concentration of dietitians located in urban, affluent areas while communities experiencing high rates of people living with overweight and obesity and type 2 diabetes mellitus are located in more disadvantaged urban suburbs and rural areas.^9^ Furthermore, one in four people with or at risk of chronic disease fail to attend in‐person consultations in community‐based and outpatient clinics, contributing to substantial healthcare resource waste.^10^ Some of the major reasons people fail to keep appointments in the community are incurring high indirect costs associated with in‐clinic consultations (e.g. time away from work, travel and costs of parking), cancellations and re‐bookings, and frustrations associated with lengthy waiting times.^10^, ^11^

Innovative health solutions can be used to create proactive, effective and sustainable services to suit growing needs and demands on the healthcare system.^12^ While these needs are recognised,^13^ historically models of care have fallen short with meeting these needs. In March 2020, the Australian Government temporarily expanded access to Accredited Practising Dietitians (APD) for Medicare Benefits Schedule (MBS) items to deliver telehealth services to Australians with an eligible chronic disease management plan, including videoconference and telephone consultations, in response to the COVID‐19 pandemic.^14^ These changes have encouraged a reframe of traditional models of healthcare delivery into virtual modalities delivered remotely that can continue well after the immediate COVID‐19 crisis.^15^

The aim of this position statement is to outline the clinical‐ and cost‐effectiveness of telehealth‐delivered dietetic consultations, and to translate this evidence to practice and policy recommendations.

## 2 SUMMARY OF EVIDENCE

This position statement is informed by a review of the existing literature reporting the effectiveness of telephone and videoconference‐delivered consultations by dietitians. The literature appraised includes recent systematic reviews where the effect of telehealth‐delivered consultations by a dietitian could be defined and compared to either a control group or a face‐to‐face consultation^5^, ^16^, ^17^, ^18^, including cost‐modelling studies.^19^, ^20^, ^21^ Further, an updated search (to April 2020) using the search terms reported in Kelly et al^6^ screened against additional criteria, including (a) telephone or videoconference diet intervention and (b) delivered by a dietitian. A meta‐analysis was performed on selected dietary outcomes. The effect of telehealth‐delivered dietetic services is arranged into five sections: (i) weight management for people who are overweight or obese; (ii) chronic disease populations; (iii) malnutrition; (iv) emerging technologies; and (v) cost‐effectiveness.

## 3 TELEPHONE‐DELIVERED DIETETIC CONSULTATIONS FOR WEIGHT MANAGEMENT

There is a growing evidence base supporting the effect of telephone‐delivered weight management services for people who are overweight or obese (body mass index (BMI) ≥25 kg/m^2^). Two randomised controlled trials (RCTs) (2013, n = 295 participants^22^; and 2011, n = 415 participants^23^) showed that weight loss in people with overweight and obesity is similar regardless of whether the dietetic consultation is delivered by in‐person mode, ad‐hoc or via telehealth.

Compared to traditional care, a recent systematic review with meta‐analysis (2019, n = 9 RCTs) concluded that telephone‐delivered weight management interventions resulted in a significant decrease in BMI for people with overweight or obesity of −0.46 kg/m^2^ (95% CI −0.73, −0.19).^18^ Recent RCTs and other study designs yield additional evidence. An RCT (2016, n = 11 participants) in people with overweight or obesity, referred by their primary care physician, found that weekly telephone lifestyle counselling by dietitians for 6 months, and second weekly calls for the following 6 months, resulted in significant weight loss. At 12 months, 47.8% of patients in the treatment group had lost 5 % of their body weight vs 11.6% in the control group (*P* < .01). The treatment group also significantly increased their moderate to vigorous physical activity compared with the control group (+126.1 minutes vs +73.7 minutes). While weight regain was observed in the 12 months after counselling stopped, physical activity was maintained.^24^

## 4 TELEPHONE‐DELIVERED DIETETIC CONSULTATIONS FOR PEOPLE WITH CHRONIC DISEASE

Telephone‐delivered nutrition care is effective for improving dietary behaviour of people with chronic diseases. Half of the existing telephone programs published in the literature are conducted in diabetes,^25^, ^26^, ^27^, ^28^, ^29^, ^30^, ^31^, ^32^, ^33^ followed by cardiovascular conditions,^27^, ^34^, ^35^, ^36^ cancer,^37^ chronic kidney disease^38^ and osteoarthritis.^39^

### 4.1 *Telephone‐delivered consultations compared to in‐person delivery*

Telephone‐delivered consultations are as effective as in‐person consultations in clinic settings.^26^, ^37^ A 12‐month RCT (2018, n = 199 participants) focused on improving type 2 diabetes mellitus self‐management behaviours, which included nutrition education once a month, led to significant improvements in glycosylated haemoglobin (HbA1c), cardiovascular disease risk and overall well‐being compared to in‐person delivery. Of note, diet quality and reduction in BMI was significant in both the telephone coaching and traditional face‐to‐face rehabilitation.^26^ Similarly, a 6‐month RCT (2016, n = 100 participants) in breast cancer survivors led to a significant improvement in dietary intake of fruits, vegetables, fibre and a reduction in fat intake with a corresponding increase in activity levels and weight loss. In this study, the in‐person weight management program was as effective, and both the in‐person and telephone coaching arm were more effective compared to traditional care.^37^

### 4.2 *Telephone‐delivered consultations compared to traditional care*

In an updated search (April 2020, n = 13 RCTs) of telephone‐delivered dietetic services, a meta‐analysis was performed that showed that telephone‐delivered consultations by dietitians was a superior intervention compared to traditional care (including those with ad hoc nutrition care) for improving a range of important dietary intake measures, including fruit, vegetable, fibre and fat intake per day (see Table 1). Telephone‐delivered consultations also significantly improved physical activity levels, reduced body weight and waist circumference, and improved cardiovascular disease risk compared to traditional care modes (see Table 1). Table 1 summarises the results of the individual meta‐analysis performed.

**TABLE 1:** Summary of telephone‐delivered dietetic services and improved diet and clinical outcomes in people with chronic disease

Outcome reported	Number of studies and participants meta‐analysed	Effect size
Fruit intake^25^, ^27^, ^30^, ^34^, ^37^, ^38^, ^39^	4 studies, 670 participants^25^, ^27^, ^30^, ^38^	MD 0.33 serves/day [95% CI: 0.18‐0.47; *I* ^2^ = 0%]
Vegetable intake^25^, ^27^, ^30^, ^34^, ^37^, ^38^, ^39^	4 studies, 670 participants^25^, ^27^, ^30^, ^38^	MD 0.53 serves/day [95% CI: 0.21‐0.84; *I* ^2^ = 0%]
Fibre intake^27^, ^34^, ^36^, ^37^, ^38^	4 studies, 1418 participants^27^, ^34^, ^36^, ^38^	MD 1.82 g/day [95% CI: 1.06‐2.58; *I* ^2^ = 0%]
Fat intake^27^, ^29^, ^30^, ^36^, ^37^	4 studies, 1418 participants^27^, ^30^, ^36^, ^37^	SMD 0.20% of total energy expenditure [95% CI: −0.31 to −0.09; *I* ^2^ = 0%
Physical activity^25^, ^26^, ^27^, ^28^, ^29^, ^30^, ^37^, ^39^	4 studies, 708 participants^25^, ^27^, ^37^, ^39^	SMD 2.54 minutes per day [95% CI: 0.71‐4.38; *I* ^2^ = 99%]
Body weight^28^, ^30^, ^34^, ^35^, ^36^, ^37^, ^38^, ^39^	7 studies, 1543 participants^28^, ^34^, ^35^, ^36^, ^37^, ^38^, ^39^	MD −1.04 kg [95% CI: −1.634 to −0.45; *I* ^2^ = 6%]
Waist circumference^28^, ^37^, ^38^	3 studies, 435 participants^28^, ^37^, ^38^	MD −2.13 cm [95% CI: −4.23 to −0.03; *I* ^2^ = 56%]
Cardiovascular disease risk^26^	1 study, 199 participants^26^	The cardiovascular disease risk reduced in telephone group, but rose in control patients (*d* = 0.12)

Abbreviations: MD, mean difference; SMD, standardised mean difference.

Combining telephone with one or more other methods of service delivery (eg, face to face; online resources, text messages, videoconferencing) produces similar outcomes to that reported in telephone‐only programs.^29^, ^34^, ^35^, ^40^, ^41^

There is conflicting evidence reported in telephone‐delivered dietetic consultations studies for some diet‐related outcomes, including diet quality^26^, ^28^, ^41^, ^42^, ^43^ and sodium intake,^30^, ^42^, ^44^ and changes in clinical variables including HbA1c,^25^, ^26^, ^28^, ^29^, ^30^ blood pressure,^25^, ^28^, ^29^, ^34^, ^36^, ^42^ lipid profiles^25^, ^28^, ^29^, ^30^, ^34^, ^36^ and quality of life.^26^, ^39^, ^42^ Larger RCTs are needed to confirm the effect of telephone‐delivered dietetic consultations for these outcomes.

## 5 TELEPHONE‐DELIVERED DIETETIC CONSULTATIONS FOR PREVENTING AND MANAGING MALNUTRITION

Telephone‐delivered dietetic counselling has been shown to be an effective method to deliver malnutrition‐related care to older adults. Malnutrition has been shown to affect up to 50% of the residential aged care population^45^ and up to 70% of hospitalised older patients.^46^, ^47^, ^48^ In a systematic review (2018, n = 9 RCTs), clinical improvements following telephone‐delivered consultation compared with in‐person dietetic care or no intervention included significantly increased protein intake, improved quality of life, and (nonsignificant) trends towards improvements in overall nutrition status, physical function, energy intake, reduced hospital readmission rates and mortality.^49^

## 6 VIDEOCONFERENCE‐DELIVERED DIETETIC SERVICES FOR CHRONIC DISEASE MANAGEMENT

Videoconference modalities to deliver nutrition care are less frequently utilised in the published literature, however, appear to be effective for managing diabetes and obesity. An Australian review (2013, n = 8 dietetic studies) of videoconference dietetic consultations concluded that these appear to be feasible and well accepted.^16^

### 6.1 *Videoconference‐delivered consultations compared to in‐person delivery*

Videoconference‐delivered nutrition care is as effective as similar programs conducted in‐person. Two of the non‐RCTs included in the review by Raven and Bywood^16^ reported on dietary outcomes, compared in‐person vs videoconference methods in people with diabetes and found clinical outcomes to be similar for a group‐based program (2012, n = 39 participants),^33^ and for a multidisciplinary (including a dietitian) individual counselling program (2011, n = 208 participants).^31^ Both these studies reported high levels of patient satisfaction, improvements in diet adherence and enhanced self‐efficacy, with improvements found in biomarkers, including HbA1c, LDL cholesterol and blood pressure.

### 6.2 *Videoconference‐delivered consultations compared to traditional care*

In clients with type 2 diabetes, videoconference interventions to deliver MNT have been shown to be more effective than traditional care (including ad hoc nutrition care), for improving a range of important diet and clinical variables. For example, the IdeaTel project was an RCT (2010, n = 92 participants) which provided 2 years of MNT and showed the group receiving videoconference counselling to have significant improvements in diet and exercise knowledge (+2.5 points compared to the control group).^32^ However, while there was significant improvement in waist circumference (by 1.2 cm over 2 years) for women, BMI and waist circumference were not significant when males were included in the overall analysis.^32^ In the only other identified RCT (2019, n = 59 participants), people with obesity received 12 weeks of telehealth nutrition coaching (which included combined videoconference and telephone consultations), resulting in significant reductions in body weight (−6.3 kg), waist circumference (−6.8 cm), and energy intake (−2520 kJ/day) and improved diet quality (+20 points) from baseline. However, the enhanced usual care (which included brief dietitian counselling) also experienced significant improvements in these measures, albeit on a smaller magnitude.^41^ Therefore, the only difference at follow up was body weight, where 70% of the intervention group lost 5% of their body weight, compared to 41% of the control arm.^41^

## 7 EMERGING DIGITAL HEALTH MODALITIES FOR TELEHEALTH‐DELIVERED CONSULTATIONS TO IMPROVE DIET AND CLINICAL OUTCOMES

Australian dietitians incorporate eHealth and mHealth technologies into their practice and patient care.^50^, ^51^ The potential of digital health to support dietitians in the nutrition care process and delivery of nutrition interventions for patients requiring weight and chronic disease management has been outlined previously.^52^ In general, patients report high acceptability, feasibility and usability for mHealth interventions targeting chronic disease management, though the technologies and implementation are not without limitations.^53^, ^54^

### 7.1 *Evidence for emerging telehealth‐delivered dietetic consultations and improving dietary outcomes*

Positive effects for food and nutrition outcomes have been observed when mHealth modalities are used for treatment and preventative service delivery. Systematic reviews report that app‐based mHealth interventions can improve dietary behaviours and intake of specific nutrients and foods, such as sodium (2019, n = 11 RCTs),^55^ vegetables, fruit, fast food or takeaway and sugar sweetened beverage intake, as well as snacking behaviours (2016, n = 27 studies).^56^ In a meta‐analysis (2016, n = 7 studies) examining e‐ and mHealth interventions for improving fruit and vegetable intakes, the outcome favoured the treatment group (pooled effect size [Cohen's *d*] 0.22, 95% CI 0.11 to 0.33; I^2^ = 68.5%).^57^A web‐based RCT (2019, n = 1125 participants) conducted in seven European countries, using personalised reports for healthy eating by dietitians or nutritionists, showed improvements in diet quality assessed by the Healthy Eating Index 2010. Improved diet quality was observed at the end of the 3‐month trial and maintained at 6 months although not all food groups' intakes improved.^58^

### 7.2 *Evidence for emerging telehealth‐delivered dietetic consultations and improving clinical outcomes*

Available evidence suggests that mHealth technologies are effective in weight management. A systematic review and meta‐analysis (2015, n = 84 studies) of web‐based interventions, mHealth interventions and other electronic communication demonstrated significantly greater weight loss in eHealth programs compared with control condition (−2.70 kg and −1.40 kg) albeit heterogeneity was present across studies.^5^ Another systematic review (2019, n = 12 studies) compared mHealth programs to either a nonintervention control or traditional dietary management and concluded that mobile apps and wearable devices are effective tools in facilitating clinically important weight loss of 5% over the duration of treatment, but these effects were not maintained at 12 to 24 months.^59^ However, overall, the evidence was limited due to only three of the 12 studies reporting results compared to a true nonintervention control group. Many interventions reported in the literature are multicomponent combining health practitioner counselling with the addition of technology such as text messaging. A meta‐analysis (2015, n = 6 RCTs) delivered via text message demonstrated significantly greater weight loss (−2.71 kg) in the intervention group compared to control.^60^

The evidence‐base supporting the effectiveness of mHealth technologies in diabetes management is growing. A recent meta‐analysis (2018, n = 17 studies) showed a mean difference in HbA1c of −0.51% (95% CI: −0.71% to −0.30; I^2^ = 47%) in groups receiving smartphone technology consultations compared with control.^61^ Another review (2017, n = 13 RCTs) showed favourable glycaemic control regardless of whether the mobile app intervention was delivered by the health professional physically or remotely.^62^

In cardiovascular disease specific literature, a systematic review (2017, n = 27 studies) of mHealth interventions identified three studies which included diet outcomes found improvements in nutrition knowledge and dietary choice with interventions that were delivered via apps, text messages and web‐based platforms.^63^ Another systematic review (2015, n = 9 studies) examined the effects of health interventions on weight loss among patients with cardiovascular disease reporting favourable outcomes for trials using web‐based platforms(−1.44 kg; 95% CI ‐2.34 to −0.34; *I* ^2^ = 98%; n = 10 studies), telemedicine (−1.04 kg; 95% CI −1.12 to −0.97; I^2^ = 0%; n = 3) and text messaging (−1.74 kg; 95% CI −2.51 to −0.98; *I* ^2^ = 83%; n = 4).^64^

## 8 ECONOMIC EVALUATION OF TELEHEALTH‐DELIVERED DIETETIC CONSULTATIONS

Telephone‐delivered nutrition programs are also cost‐effective. When compared to the same weight management program delivered face‐to‐face over 18 months, telehealth‐delivered programs were more cost effective (2013, n = 295 participants).^22^ Further, an in‐person group‐based obesity management RCT in rural settings (2012, n = 215) showed telephone counselling resulted in a lower cost per kilogram weight loss (AUD 52.50/kg) vs face‐to‐face (AUD 74.77/kg).^65^ An RCT (2016, n = 111 participants) in a Brisbane hospital outpatient setting found individual telephone counselling was more effective than a group based in‐person program and the cost per healthy life year gained was AUD 33000 and AUD 85000, for the telephone and group program, respectively.^66^

In chronic disease studies specifically, comparing telephone‐delivered nutrition consultations to usual care (including those with ad hoc nutrition care), four of five interventions were found to be cost‐effective^38^, ^67^, ^68^, ^69^ in people with diabetes, hypertension, chronic kidney disease and people undergoing cardiac rehabilitation. However, the intervention in one of the five studies conducted in osteoarthritis patients was not cost‐effective when compared with usual care.^70^

For cost‐effectiveness of emerging telehealth interventions, a systematic review (2020, n = 23 studies) in type 2 diabetes reported mHealth interventions were highly cost‐effective, with cost per Quality Adjusted Life Years (QALY) gained ranging from 0.4% to 62.5% of GDP per capita. The costs varied depending on the number and type of technologies employed that ranged from one technology to three.^71^

## 9 IMPLEMENTATION OF TELEHEALTH‐DELIVERED DIETETIC SERVICES

An existing practice‐based evidence in nutrition (PEN) knowledge pathway is available for APDs, which includes practice points for delivering telephone consultations for adults with chronic disease, non‐chronic disease management telephone programs and telephone interventions for improving nutrition outcomes in infants and new mothers.^72^

One of these PEN knowledge pathways highlights the lack of evidence for call centre support for public health nutrition interventions and government policy implementation, which is due to a lack of evaluation studies in the published literature.^72^ However, there are existing telehealth programs with nutrition components in Australia, but these are not always specific to dietetic services. For example, since 2009 NSW Health has offered the community Get Healthy Coaching and Information service which provides 10 telephone‐delivered coaching sessions over 6 months aiming to improve nutrition, physical activity and, if desired, weight loss. The first evaluation of the service (2014, n = 1440 participants) revealed significant weight loss of 3.9 kg, increased fruit and vegetable intakes and physical activity with decreased intake of take‐away meals and sugar sweetened beverages.^73^ Since then, there have been telephone coaching services offered to different population groups that have been evaluated including Aboriginal and Torres Strait Islander people (2017, n = 103 participants) showing a significant mean weight loss of 3.3 kg,^74^ those at risk of type 2 diabetes (mean weight loss of 3.3 kg, *P* < .001, n = 4442),^75^ and a pilot program (2019, n = 89 participants) in pregnant women to avoid excessive weight gain, showing a nonsignificant difference of 42.9% in the coaching program vs 31.9% in the control meeting recommended weight gain.^76^

Conceptual models for effective telehealth within chronic disease management have been proposed.^77^ Success factors in implementing a telehealth model identified by O'Cathain and colleagues include ensuring that both the human and technical aspects of telehealth operate well. These implementation considerations are summarised in Table S1. Dietitians Australia has highlighted suitable candidates for telehealth dietetic services.^78^ These suitable candidates and practical strategies to be considered for optimising telehealth outcomes are also summarised in Table S1.

By considering factors specific to delivery of virtual nutrition care by videoconference, dietitians can use their expertise to deliver services that complement, rather than compete with existing and emerging technologies. Issues specific to using videoconference in dietetic service delivery can be addressed through use of a checklist to support them during delivery of MNT in order to facilitate effective and efficient virtual nutrition care.^79^

Substituting telehealth services for standard consultations covered by MBS Item 10954  would be cost neutral for the consultation. Advice from the Department of Health is that patients accessing chronic disease management MBS items claim an average 2.5 allied health (not dietetic‐specific) items per year. Expanding access to telehealth‐delivered dietetic consultations will result in improved outcomes which would reduce expenditure on medications and decrease hospital costs as demonstrated by the pilot of the Diabetes Care Project.^80^ Any increase in the number of consultations for dietitians may not require an increase in the health budget but more sophisticated analysis of the current pattern of usage of chronic disease management MBS item numbers to allow modelling of potential changes in its usage.

Appropriate and effective use of technology within practice is a key competency standard outlined in National Competency Standards for Dietitians in Australia.^81^ Dietitians possess all the skills required to provide MNT using telehealth. Taking courses in eHealth either as part of dietetic training, or as continuing professional development for APDs, can improve the understanding of concepts essential for using telehealth and eHealth technologies.^82^ Key components include definitions of eHealth terms and concepts related to telehealth and mHealth technologies; and knowledge and skills related to (i) use of telehealth equipment, (ii) comparison of dietetic consultation components completed in person vs remotely via video call, (iii) quality assessment of mobile apps and (iv) exploration of advantages and disadvantages, and the ethical, security and privacy issues relating to use of eHealth technologies in dietetic practice. This training and professional development in delivery of nutrition and dietetic consultations using telehealth results in improved knowledge, skills and competence in using these technologies.^82^, ^83^

## 10 FUTURE RESEARCH OPPORTUNITIES

There are a number of opportunities for further research concerning telehealth‐delivered consultations. Specifically, clinical trials are needed to evaluate the implementation of telehealth consultations delivering group‐based interventions in populations with chronic disease, and improving access and outcomes for vulnerable populations groups, including those in regional and remote areas through telehealth‐delivered consultations. There is also a need to understand the challenges of completing some components of nutrition care via telehealth (e.g. physical measures) and evaluate alternative or modified measures to recommend as suitable proxies.

Robust economic evaluations are needed across different chronic disease populations and demographics which are most likely to benefit from wider access to dietary services under Medicare, including rural/remote areas and house‐bound individuals. An economic evaluation should also consider and evaluate the societal benefits of telehealth‐delivered consultations that cannot always be captured by typical economic analysis using a healthcare perspetive,^20^, ^21^ including willingness‐to‐pay (ie, evaluating the monetary value on the benefit associated with a service, from a societal perspective), and any unintentional consequences that new dietitian delivered telehealth consultations may potentially have (e.g. consequences which may arise from unexpected uptake, creating inequity for populations that may not have access to technology hardware or reliable phone or internet service due to financial disadvantage, which substantially increases costs, unexpected workload changes or other unforeseen factors).

Finally, it will also become important to evaluate the effectiveness of emerging technologies including mHealth and eHealth nutrition programs alone, in combination with telephone or videoconference programs, or when combined with in‐person delivery to reduce the number of counselling sessions required. These evaluations, in addition to addressing the evidence gaps mentioned above, will allow decision makers to make informed, evidence‐based decisions on telehealth‐delivered dietetic consultations.

## 11 RECOMMENDATIONS

The summary of results presented in this position statement support the evidence‐based recommendations summarised in Table 2.

**TABLE 2:** Evidence‐based recommendations for telehealth‐delivered consultations

Practice area	Recommendation
Weight management	Telephone counselling is effective for management of overweight and obesity in primary care.
Chronic disease management	Telephone and videoconference nutrition consultations improves diet, physical activity levels and reduces body weight in people with chronic conditions. Telephone and videoconference consultations are just as effective as in‐person delivered MNT.
Malnutrition	Telephone counselling is effective for the prevention and management of malnutrition in the community.
Digital health	Digital health solutions (including eHealth (e.g. web platforms) and mHealth (e.g. smartphone applications)) can support traditional in‐person or telephone and videoconference delivered nutrition care, but their effectiveness as a delivery modality exclusively requires further research.
Funding for telehealth‐delivered dietetic services	Government policy makers and healthcare funders should broaden remuneration benefits for telephone and videoconference‐delivered consultations provided by APDs, as these are cost‐effective and low cost for APDs to operate. Expanded telehealth access under Medicare and Private Health payers addresses health and service inequalities, improves access to effective nutrition services, and supports people with chronic conditions to optimise their diet‐related health and well‐being, regardless of their location, income or literacy level. MNT delivered via mHealth and eHealth should be considered eligible for Medicare or Private Health rebates when they are used alongside telephone or video conferencing modalities or in‐person delivery.

Abbreviations: APD, accredited practising dietitian; MNT, medical nutrition therapy.

## CONFLICT OF INTEREST

This position paper was commissioned, funded and endorsed by Dietitians Australia. Funding supported the lead author to conduct the literature searches and draft the paper.

KC and TD are members of the Dietitians Australia board. Neither received any specific funding from Dietitians Australia throughout this project.

## Supporting information


**Table S1** Strategies for optimising telehealth outcomes adapted from Salisbury et al^82^ and Dietitians Association of Australia^83^
