# Supplementary material for: Dietitians Australia position statement on telehealth
Source: Nutr Diet. 2020 Jun 28;77(4):406–15. doi: 10.1111/1747-0080.12619 (PMC7540717; doi:10.1111/1747-0080.12619)
Supplement: Supplementary file 1 — Table S1 Strategies for optimising telehealth outcomes adapted from Salisbury et al82 and Dietitians Association of Australia83 [file NDI-77-406-s001.docx]

**Supplemental Table 1.** Strategies for optimising telehealth outcomes adapted from Salisbury et al^82^ and Dietitians Association of Australia^83^

| **Strategy to optimise with telehealth**^82^ | **Considerations for dietetic practice**^83^ |
| --- | --- |
| **Patient and health professional engagement** | - Ensure appropriate equipment and location in which to consult (for both Dietitian and client/patient) - Administrative support to manage attendance, assist with providing nutrition resources as directed by the dietitian - Patients actively wanting the service receive it and that staff delivering the service are enthusiastic, motivated and provide continuity of care and delivery^89^ - Encouraging patient activation and involvement rather than passive reminders   **Additional support may be required for:**   - Clients who are hard of hearing or who have a disability may need assistance from a support person - Patients with poor English may require the assistance of a translator who may be present or dialled in by telephone |
| **Effective chronic disease management** | - Optimise strategies for self-management, treatment optimisation, and coordination of care - Potential to deliver care at an increased frequency, known to support behaviour change |
| **Provider partnerships and human aspects of telehealth** | - Active engagement with primary care staff involved in patient is strongly encouraged, so all members of the multidisciplinary team understand the recommendations provided to reduce confusion among patients and care providers^89^ |
| **Consideration of the patient, social and health system factors** | - Prioritise elderly, frail, have a disability or have work, personal, caring or family responsibilities that make attending an appointment difficult - Those who might need to see a dietitian urgently - Those who would need to travel for many hours (or even days) to attend an appointment - Those who are seeking a dietitian who works in a particular practice area - Those who would prefer a telehealth consultation |
